# Complicity at a distance: commemorating problematic involvement in perpetration in contemporary Central and Eastern European literatures

**DOI:** 10.12688/openreseurope.14631.2

**Published:** 2022-05-25

**Authors:** Juliane Prade-Weiss

**Affiliations:** 1 Department of Comparative Literature, Ludwig Maximilian University of Munich, Munich, Bavaria, 80799, Germany

**Keywords:** complicity, postmemory, mass violence, genocide, transgenerational trauma

## Abstract

**Background**: In the twenty-first century, literatures from Central and Eastern Europe are marked by a boom of documentary fiction portraying complicity Nazi perpetration, Soviet terror, or other instances of 20th century mass violence and totalitarianism. Since understanding the past serves requirements of the present, the boom prompts the question: Why the interest in past complicities now? My hypothesis is that the texts address convergences between involvements in past acts of mass violence and current forms of participation in wrongdoings in neoliberalism. While these issues differ profoundly, they are related: structurally, both present the challenge of forming a nuanced notion of participation. Historically, they are related since justifications of past involvements have established the terminology, narratives, and heuristics in which terror, repression, and mass violence are subsequently discussed, thus forming the frame for negotiating current problematic involvements.

**Method**: Critical discourse analysis is used to scrutinize the legal concept of complicity and combined it with close readings of passages from four literary texts to outline how attention to reciprocity in language can enhance our understanding of problematic involvement.

**Results**: Literary portrayals of historical complicity are ambivalent; they can help to find models for comprehending issues of the present in cultural memory, but they can also serve to establish distance between present and past to appease the sense that all is not quite well, even after the demise of Nazi and Soviet terror. The article outlines two modes of distancing: a) spacio-temporal distancing of the commemorating point of view in ‘the West’ from the portrayed violence in ‘the East’, and b) moral distancing that casts the audience as superior to complicit characters.

**Conclusion**: By pressing for analytic or consoling distance, both strategies of distancing amount to a complicity with the transmission of discourses that justify, excuse, or deny mass violence and totalitarian terror.

## Plain language summary

In the twenty-first century, many literary texts from Central and Eastern Europe portray complicity of characters with political violence such as National Socialist crimes and Soviet terror. I analyse texts written in German, Czech, and Russian which combine fact and fiction; they portray historical events but make use of fictional characters or dialogues. The article seeks to answer the question: why are is historical complicity currently portrayed? Why should it matter to current readers? My assumption is that what makes such texts popular is that reading about what might have driven people in the past to get involved in political violence and terror can serve to understand how and why people are currently involved in wrongdoing, for instance in economical injustice or ecological damage. Taking part in structures we know are wrong seems hard to avoid in a globalized world, and this sentiment can also be found as a dilemma of people who lived in oppressive regimes of the past. I inquire into the legal understanding of complicity and outline that the law is not enough to understand how people get enmeshed in wrongdoing. I show that some literary texts which portray historical complicity can contribute to understanding what makes people take part in wrongdoing because they show the subtle pressures, emotions, and personal relations that cannot be addressed in the courtroom. However, my article further shows that some literary texts follow a different agenda and seek to distance current readers from violent pasts so as to console them about their involvement in current wrongdoing. I argue that what makes fictional portrayals of actual historical events relevant (even if they are not true) is that they draw the readers’ attention to the fact that how we commemorate violent pasts is decisive for how we understand our role in current violence.

## Introduction

In the twenty-first century, literatures from Central and Eastern Europe have been marked by a boom in testimonies of involvement in twentieth century mass violence ^1^ and totalitarianisms. Texts such as, for instance, Radka Denemarková’s *Peníze od Hitlera* (2006; Money from Hitler, 2009), Elfriede Jelinek’s *Rechnitz (Der Würgeengel)* (2008; trans. Rechnitz [The Extermination Angel] 2015), Herta Müller’s *Atemschaukel* (2009; trans. The Hunger Angel, 2009), Jáchym Topol’s *Chladnou zemí* (2009; trans. The Devil’s Workshop, 2013) and Maria Stepanova’s *Памяти памяти* (2017; trans. In Memory of Memory, 2021) portray complicity with Nazi perpetration, Soviet terror, or other instances of twentieth century mass violence and totalitarianism in contemporaries and descendants. Since understanding the past always serves requirements of the present, the boom prompts the question: why the interest in past complicities now?

The comprehensive background of my reading is the hypothesis that the texts address convergences between involvements in past acts of mass violence and current forms of participation in wrongdoings of humanitarian, political, ecological, or other natures in neoliberalism. While these issues differ in many respects, they are related in structural and historical terms. Structurally, both present the challenge of forming a nuanced notion of participation, the idea and promise at the heart of democracy, digital media, and consumer capitalism that is highly valued yet poorly conceptualized. Historically, both issues are related since justifications of past involvements have established the terminology, narratives, and heuristics in which terror, repression, and acts of mass violence are subsequently discussed by inscribing them into cultural traditions, thus forming the frame for negotiating current problematic involvements. The convergence is, therefore, of particular interest in view of the global crisis of political participation, which is currently undermined by an often unwilling but inevitable participation in detrimental economic structures that can be linked to the ecological crisis, the delegitimization of democracy, and the retreat to identitarian ideologies, not least in “memory wars.” ^2^ The modes of complicity stand out more clearly—and are acknowledged more readily—with reference to past violence. They are more complicated, but just as active, in the globalized world of the present.

The segment of this wider context that will be discussed within the confines of this article pertains to the stance of the present outlook onto the past: some texts highlight distance from the violent past while others emphasize the onlookers’ involvement in an ongoing transmission of the aftermath of past violence. This duality arises from a profound ambivalence in the analogy between totalitarianisms and neoliberalism. Complicities in past totalitarianisms may be paralleled with current problematic involvements to find models for comprehending issues of the present in cultural memory and/or to understand the genealogy of forms of social interaction and their justification. This analytical approach is counteracted by hedonistic, or consoling, readings which evoke instances of past complicities to appease the sense that all is not quite well, even after the demise of Nazi and Soviet rule, by drawing attention to how bad, how much worse things have been, and grant distancing. This effect has been studied in German mass media representations of the Shoah, which, as Giesen points out, create a “collective memory” by way of “identification with the past” at the price of permitting to “consume this disconnected past as exotic alterity and even as sentimental entertainment.” ^3^ Authorial intent cannot prevent such readings. Literature differs eminently from juridical discourse—from which the term *complicity* is borrowed—in that authorial intent is not decisive for the reception of a text. What matters is the complex relation between identificatory options offered by the text and the readers’ various ways of adopting them. This relational openness is decisive especially in the genre of documentary fiction, to which most of the texts portraying historical complicities belong.

Documentary fiction, i.e., texts portraying historical events in a fictitious plot or dialogue give accounts of acts of mass violence that evade conventional historiographic means, because of a lack of surviving victims who could testify as well as political and psychological resistance in perpetrators, accomplices, and descendants. Therefore, Madigan notes, “literature often plays an outsized role in its ability to represent and broadcast trauma at the cultural level”. ^4^ While the conflation of fact and fiction is prone to raise ethical concerns, ^5^ non-factual accounts are indispensable to shaping a collective memory of forms of violence that aim at exterminating groups of people and their cultural heritage. ^6^ I speak of “documentary” rather than “historical” fiction because a pivotal concern of the texts is how documented historical facts are merged into both societal historical narratives and personal memory. The purpose of documentary fiction is neither to forge historical facts or even to convey facts, nor to form juridical decisions, but to confront audiences intellectually and emotionally with complex situations of ethically problematic involvement. Works of documentary fiction rely on the fact that all reading is based on participation, as texts speak to implicit readers, and that literature requires the participation of audiences, be it the voice and imagination of the reader or the gaze of the spectator. Fiction, moreover, depends on what Coleridge calls a “willing suspension of disbelief”. ^7^ Documentary fiction relies on reader participation to reflect on instances of historical participation in mass violence. The point of this aesthetic reflection is neither to prove the readers’ distance from a ‘tragic past’, nor to rule out such readings, but to draw attention to exactly the issue of distance—be it hedonistic or analytical. Both imply emotional distancing, while reading requires participation.

In the following, I will juxtapose two literary texts in order to analyze two prominent alleys of distancing that constitute the very opposite: not distance, but a particular form of complicity with the transmission of discourses that justify or foster mass violence. These modes of distancing are relevant because they are no mere literary phenomenon but mirror elements of the wider socio-political debate on memory culture. Some texts reproduce these modes of distancing, others reflect on them. One mode is a spatio-temporal distancing of the commemorating point of view ‘in the West’ from the portrayed violence in ‘the East’. It will by outlined by way of reading passages from Valerie Fritsch’s 2020 novel *Herzklappen von Johnson & Johnson* (Heart Valves by Johnson & Johnson). A second strategy, used particularly when spacial distancing is impossible, is moral distancing, i.e., to cast the narrator, and readers, as morally and/or intellectually superior to complicit characters. This strategy is discussed in Elfriede Jelinek’s 2008 play *Rechnitz (Der Würgeengel)* (trans. Rechnitz [The Extermination Angel], 2015). To elucidate the convergence between past complicities and involvements in neoliberalism, I will, furthermore, refer to Maria Stepanova’s 2017 essayistic novel *Памяти памяти* (In Memory of Memory, 2021), which I have discussed in detail elsewhere. ^8^ Outlining the two strategies of distancing from the violent past, however, first of all, requires a brief discussion of the concept of complicity because its complications in juridical discourse are what renders it productive in literary texts.

## Complicity in and beyond the Law

As a legal term, complicity describes the way a crime is committed, namely by aiding or abetting wrongdoing. Yet complicity poses a challenge to the law as it undermines the principles of individual accountability and autonomous action: dependent on the actions of a principal wrongdoer, the accomplice is still autonomous insofar as aiding or tolerating wrongdoing makes a difference. ^9^ Accountability is based on individual intentionality, which gives rise to a particular difficulty in current corporate and international law, whereby corporate and state complicity with human rights infringement and environmental damage often evades sanction, because corporations and states are not understood to have intentions. This, paradoxically, renders them actors without intent. ^10^ Complicity thus marks the limits of legal discourse by pointing beyond the law’s methodological individualism to structures of social relationality. This connectedness is exploited in the totalitarian strategy of reassuring the individual’s sense of guilt while undermining individual action and personal accountability—a process outlined in Arendt’s maxim, “Where all are guilty, nobody is”. ^11^ Declaring everyone guilty is tantamount to labelling wrongdoing inevitable and—in ultimate analytical complicity—to dropping the differentiation between moral choices, just as it had been aborted in totalitarianism. 

To move beyond methodological individualism, legal research proposes the evaluation of the causal contribution to wrongdoing independent of intent ^12^, or notions such as “shared responsibility” ^13^ and a “participatory conception of collective action”. ^14^ What participation means, however, is a second challenge complicity entails in and beyond legal thought because despite its popularity in political philosophy and popular parlance, participation is a defined concept neither in Leibnitz, Kant, Fichte, nor in Schelling, Hegel, Nietzsche or other authors of classical Modern thought. ^15^

This issue is where literary discourse can make a decisive contribution. Assessments of historical involvement in wrongdoing rely on reconstructive narratives ^16^, and conceptual analyses are based on hypothetical scenarios. ^17^ The structure of these narratives, however, the relation between fictional, documentary, and prescriptive legal speech is hardly reflected. Yet the medium of language is, in fact, a good model for approaching the complication of complicity, that is: individually responsible participation in a communal structure. Responsible individual speakers cannot but use preformed phonetic, semantic, and syntactic structures. The relational aspect of human action that poses a problem to legal thought is the focus of literary discourse, which foregrounds language as principal medium of human interaction. This is as true for the spoken word as for texts. In analyzing literary discourse, the notion of complicity is useful not because it provides clarity (it does not), but because it marks problematic participation, enmeshment, and degrees of responsibility that may evade straightforward legal culpability. ^18^

One such instance of problematic implication relates to the very concepts “totalitarianism” and “neoliberalism.” Both are highly politicized and often employed for polemical rather than analytical purposes. Just as complicity, the terms totalitarianism and neoliberalism are relevant for a discourse that reflects on the point of view of the commemorator as inscribed in conventions of cultural memory because they cast doubt on the claim to analytical distance. Totalitarianism is a historiographical concept to describe a particular form of power that controls all aspects of life by way of a mixture of utopianism, scientism, and political violence. ^19^ The term was adopted by Italy’s Fascism and Germany’s National Socialism, became a discursive weapon of the Cold War, ^20^ and a rhetorical stopgap in the present. ^21^ The same heterogeneity holds true for the economic term neoliberalism, “a rather broad and general concept referring to an economic model or paradigm that rose to prominence in the 1980s” and comprises heterogeneous elements: an ideology, a form of governance, a policy, and a form of capitalism. ^22^ “Neoliberalism” has become a notorious catchphrase for criticizing the 21st century state of affairs, yet the term has always been politicized inasmuch as it has been conceptualized as an ideological counterpart to totalitarianism. ^23^ It proves to be impossible to comprise all current strands and theories of neoliberalism in one definition, yet what is relevant here is that historically, neoliberalism is a post-World War II response to the “crisis of liberalism” brought about, in large part, by European totalitarianisms. ^24^ There are, of course, substantial “differences between Fascist and Communist regimes”, but “according to the neoliberal view their common denominator is that they are collectivist” ^25^, while neoliberalism seeks to maintain “’an individualist civilization’”. ^26^

While this paper cannot do full justice to the debates on totalitarianism and neoliberalism, it seeks to highlight two discursive phenomena. Firstly, theoretical concepts participate in historical processes as much as they describe them. Concepts may be regarded as archives of cultural memory ^27^ since their implications and incoherence testify to socio-political ruptures and consequent hermeneutical crises. ^28^ Secondly, while such incoherence complicates exchange and fosters polemic, it is also what permits the accommodation of different voices and divergent positions in social interaction. This is to say that while the claim to analytical distance enables an important form of discourse, namely critique, theoretical language does not grant a position outside the discursive parameter of cultural memory but enables speakers to participate in discourse. A striking case in point is the analogy between totalitarianism and neoliberalism that has been drawn, for instance, in political science, to criticize the idea of a spontaneous market order as bearing totalitarian markings as it equals self-regulated economy with civilization. ^29^ Zuboff criticizes the parallel as inappropriate for understanding digitalized “surveillance capitalism”, and cites the belated understanding of Nazi and Stalinist totalitarianism in 20th century contemporaries as “vivid precedent for this kind of encounter with an unprecedented new species of power.” ^30^ While it is true that the concepts of totalitarianism and neoliberalism describe very different phenomena, paralleling them is, still, no fallacy but a conventional hermeneutic strategy of cultural memory: transferring testimonies of past experience to unprecedented purposes. Literary texts are a key medium of this transfer, particularly documentary fiction that negotiates the stance of the present onlooker towards past complicities in mass violence and totalitarianism.

Common sense and neoliberal theory would certainly locate totalitarianism and neoliberalism on opposing ends of the political spectrum. This is doubtlessly true in terms of the ideas underlying these political orders, yet the polarity does not hold utterly true in terms of human relationality. I speak of a convergence between—rather than a similarity of—complicities with totalitarianisms and involvements in neoliberal wrongdoing to point out that while these orders are certainly different, individual experience in both shares in a problem. Narratives of complicity are, of course, no recent phenomenon, ^31^ yet the current popular fascination with the issue appears to be indicative of a particular modern urgency in thinking about willing and unwilling participation and shared agency. An important clue about the background of this fascination can be taken from Arendt, who writes about twentieth century totalitarianisms:

*[F]reedom from politics […] was unknown in antiquity, and it has been quite effectively abolished in a number of twentieth-century dictatorships, especially of course in the totalitarian variety. In contrast to absolutism and other forms of tyranny, where nonparticipation was a matter of course and not of choice, we deal here with a situation where participation, and that as we know can mean complicity in criminal activities, is a matter of course, and nonparticipation a matter of decision. ^32^*

That “nonparticipation” is “a matter of decision” holds true for the neoliberal present of globalized markets, conflicts, climate change, and digital media: the individual maybe conceptualized as free to decide not to participate in them, yet implementing this decision is hard, and often comes at the substantial prize of non-inclusion into key societal structures. More abstractly speaking, what makes complicity a ubiquitous phenomenon in (not only Western) Modernity might be the parallel of a far-reaching striving for civic participation of individuals in mass-based societies on the one hand, and the lack of sophisticated political concepts of participation outlining the relationality of the so-called Modern individual on the other. This is, yet again, where the relevance of literary texts to the broader societal discourse comes in. Because rather than insisting on analytical distance, as terminological language has to, literary texts can explore language as principal medium of human relationality and point out that insisting on distance from the problematic past involvement of others misses the seminal, albeit uncomfortable, affectedness of the observer that is the very basis for analysis and critique.

Complicity, this brief exploration has shown, is an ambivalent charge as it is hardly functional within and too general outside a legalistic framework. Complicity can, however, be analytically productive if used not as a charge but as a marker for complexity. Analysing the role of intellectuals in South African apartheid, Sanders distinguishes “acting-in-complicity,” which can be legally and ethically judged, from an underlying “responsibility-in-complicity,” a connectedness with others that explains why even silence, non-listening, or inactivity may affect the lives of others. ^33^ I suggest taking responsibility literally to examine how texts *respond* to the connectedness with others and their critical role in transmission.

## Spatio-temporal distancing

Due to their peculiar possibility of merging fact and fiction, literary texts can address and counteract repression and denial in ways other discourses cannot. However, since literature is part of societal discourse, literary text just as often reproduce strategies of constructing a position of reading untouched by involvements. In portrayals of complicities with mass violence and totalitarianisms in Europe, a crucial mode of maintaining the non-involvement of the present observer is drawing a stark distinction between the present position of the author, and the reader, in the West via-à-vis the past violence as belonging to the East.

The distancing is satirised in Topol’s *Chladnou zemí* (The Devil’s Workshop), which portrays a globalized industry of complacent popular memory politics at the Theresienstadt (Terezín) Ghetto, one of the sites of an industrialized genocide. And Topol summarizes the hierarchy imposed by the current spatio-temporal distancing from mass violence and complicity: nobody, his text suggests, wants to situate themselves in ‘the East’, not even a character speaking in Vladivostok, the Pacific railhead of the Trans-Siberian Railway: *Jakej Východ, […], zbláznila ses? Tady je přece Západ, opravdickej konec Západ, tady je konec Evropy!* ^34^ ( *—*“East, … are you crazy? Why, this is the West, the honest-to-God end of the West, this is the end of Europe!”) ^35^ In absurd mimetic participation in a discourse that defines itself as Western, the East self-eliminates.

What appears absurd in Topol’s 2009 text was mirrored in political rhetoric ten years later, when president of France Emmanuel Macron stated, at a 2019 press conference held together with president of Russia Vladimir Putin, at Fort Brégaçon: *nous croyons dans cette Europe qui va de Lisbonne à Vladivostok* (“we believe in this Europe that goes from Lisbon to Vladivostok”). ^36^ In this gesture of outreach, the representative of Western Europe rhetorically incorporates a large part of Asia into Europe, and implicitly eliminates any non-Western point of view.

This outreach, which Topol satirises, constitutes a bona fide poetics of nostalgia in Fritsch’s 2020 novel *Herzklappen von Johnson & Johnson*, which portrays Eastern Europe as a hardly inhabited landscape of transgenerational trauma. Fritsch, an Austrian author, tells a story of the silence of perpetrators and its transmission onto the second and the third generation of descendants. If the narrator’s grandfather speaks at all, he tells his story of “the war” ^37^, World War II.

*Sie klang immer falsch und war so verwirrend, dass man die Opfer und die Täter verwechseln konnte […] und klang, als wäre der Großvater kein aktiver Teil davon gewesen, als hätte er die beschwerlichen Zeiten nicht selbst gelebt und als wäre ihm der Krieg, der immer noch nicht richtig zu Ende schien, bloß zugestoßen*.
“It always sounded wrong and was so confusing that you could mistake the victims for the perpetrators. […] and [it] sounded as if the grandfather had not been an active part of it, as if he had not lived through the difficult times himself and as if the war, which still did not seem to be over, had just happened to him.” ^38^

The grandfather’s narrative distancing is mirrored in the narrator: initially, she fills in the emotional void in his place by way of identification. The narrator enjoys the sad wartime stories as “surrogate pain, a vague substitute ache”— *Ersatzschmerz, ein unbestimmtes Stellvertreterweh* ^39^—which fills the void left by her ancestors’ silence. She thus dreams of the destructions her grandfather may have caused during the war and of his suffering as a POW in a camp in the Kazakh steppe:

*Weite Ebenen voller Menschen in einem Augenblick und leer im anderen. Von baumlosen Landschaften, deren Weite und Hoffnungslosigkeit einem ins Herz schnitt*. ^40^
“Vast plains full of people one moment and empty the next. Of treeless landscapes whose vastness and hopelessness cut into one’s heart.”

These dreams are ambivalent: nightmares at first, they later give rise to the longing for actually seeing the places he had seen.

*Sie […] begann davon zu träumen, weiter und weiter zu fahren, weiter in die Vergangenheit und weiter in den Osten, bis in den Krieg hinein, bis in die Gefangenschaft des Großvaters, bis in die kasachische Steppe. […] Sie wusste, dass es ein vermessener Wunsch war und ein maßloser, eine unmögliche Zeitreise, aber sie störte sich nicht daran*. ^41^
“She […] began to dream of going further and further, further into the past and further east, into the war, into grandfather’s captivity, into the Kazakh steppe.
[…] She knew it was a presumptuous wish and an immoderate one, an impossible time travel, but she was unbothered by this.”

In fact, both the narrator and the narrative presentation remain unbothered by the self-indulgent nature of the wish to bridge the spatio-temporal gap between her, and her grandfather’s experience. With the journey, the narrator’s unsettling lack of distance from his sequelae of mass violence perpetration is translated into a reenactment of his distancing. The catalyst of this transmission is the assignment that sets the journey in motion: the narrator’s partner, a photographer, is commissioned to find images of post-communist ruins:

*eine große Bilderstrecke über verfallene Bauwerke und Industrieruinen in den Ländern des Ostens zu photographieren, von der Ukraine bis nach Aserbaidschan.* ^42^
“to shoot an extensive photo spread of dilapidated buildings and industrial ruins in the countries of the East, from Ukraine to Azerbaijan.”

They find exactly what they are looking for, the ruins of traumatized landscapes:

*Auf ihrem Weg trafen sie auf verlassene Bauernhäuser, aufgelassene Tankstellen und aufgegebene Dörfer. Halb eingestürzte Kirchen, in denen Singvögel auf den morschen Bänken nisteten, kleine Gotteshäuser […] ohne Kreuz, ohne Gott. Industriekathedralen, kühl und still. […] und oft schienen ihnen nicht nur die Orte verlassen, aber auch die Menschen am Straßenrand, an denen man vorüberfuhr. Manche wirkten verfallen wie Häuser.* ^43^
“On their way they encountered abandoned farmhouses, abandoned gas stations and abandoned villages. Half-collapsed churches, where songbirds nested on the rotten pews, small chapels […] without a cross, without God. Industrial cathedrals, chilly and silent. [...] and often not only the places seemed deserted to them, but also the people on the side of the road that one passed. Some seemed derelict like houses.”

Although the journey leads to the place of the grandfather’s imprisonment, there is nothing to be found, as the text states, “nothing that would have sufficiently testified to the past.” ^44^ One place, however, stands out in the journey that hardly identifies cities or landscapes: the forest of *Bronitza* (Брониця, Bronica) near the Ukrainian town of *Drohobytsch* (Дрогобич, Drohobych). ^45^ The fact that it is singled out suggests that this place also stood out in the grandfather’s stories. Fritsch does not give any historical background, therefore, I do: in 1941, with the Nazi invasion of the Soviet Union, a ghetto was established in Drohobych, from where Jewish inhabitants of the town and the region were deported into concentration camps; the ghetto was dissolved in 1943, and the remaining prisoners were massacred in the nearby forest of Bronica. ^46^ In Fritsch’s text, however, the name of the place stands out just as traumatic experience remains unconnected to other, previous and later, experience.

Fritsch’s text has been met with great critical acclaim for its poetic qualities and portrayal of the transgenerational sequelae of perpetration. ^47^ This portrayal, however, is problematic since the text never reflects on the fact that the landscape traversed by the narrator is a geographical display of her heritage of silencing, emotional emptiness, and emphasis on non-involvement. Fritsch’s ruined East is a soulscape, as it were, leaning on the Romanticist aesthetic insight that what renders an area a *landscape* is that it mirrors the onlooker’s emotions. Despite this pedigree, the nostalgic image of a ruined Eastern Europe is problematic because exoticist projection comes at a price, as has been pointed out with reference to other global instances. Looking at the East to find nothing but the ruined heritage of Nazi perpetration and denial forestalls any dialogue with people who inhabit this realm. Acting out the ancestors’ suppressed emotions and, as a consequence of this identification, reenacting their distancing from the violated realm, amounts to a complicity with past perpetration inasmuch as it maintains one-sidedness and transmits the point of view of perpetration.

This is, of course, not to vouch for the anthropological accuracy of literature. Basis of my criticism is that as literary texts appeal to audience imagination by citing common assumptions (like Topol cites the expectation that nobody wants to be located in the East), literary texts carry a certain responsibility. What is crucial is how they *respond* to the issues they address. This response is missing in Fritsch’s text, which portrays the logic of the transgenerational transmission of the sequelae of mass violence perpetration neatly along the lines of what humanities and social sciences research has outlined, but aspires to no reflexion on, or aesthetic disruption of, its logic beyond the portrayal of unhappy self-absorption. And the pleasure of reading the text shares in the enterprise of remembering to forget—in the sense that engaging with a “’comfortable horrible’ memory” ^48^ may reassure audiences of a responsible outlook while diverting attention from the ethical complications it entails. In this setting, engaging with the past turns into a form of consumerism that reduces the awareness of sequalae of terror and violence to symbolic capital, to a token of the commemorator’s social responsibility. The content of this token is replaceable but not arbitrary, as concern for past violence stands in for the regard for current forms of political and institutional violence or economic injustices, which are more uneasy to engage with because observers may find themselves complicit with them.

It has been argued that “[t]he formation of neoliberalism and the rise of memory are two strictly contemporaneous phenomena,” and that this is no coincidence: Unlike a grand récit of global progress and liberation, “[f]ragmented and subjective memories do not challenge the existence of capitalism”. ^49^ Focusing on family memory rather than historical utopias can thus tie in with neoconservatism which, in turn, goes well with neoliberalism due to its insistence on the status quo. ^50^

Fritsch’s poetics of traumatic projection elucidates a further aspect of Macron’s political projection of Europe all over the Asian part of Russia: both do not reckon with any agency positioned in the ‘Eastern’ realm. Against the backdrop of Russia’s 2022 invasion of Ukraine, which ties in with the expansionist policy of “Russian World” (Русский мир), ^51^ it becomes clear that Putin may have agreed with Macron’s 2019 expansionist outreach, uttered in the first-person plural, yet with a very different agenda in mind. Reducing ‘the East’ to a canvas for traumatic projection or political projects from a point of view which understands itself, by contrast, as ‘Western’, precludes one from making encounters and thus renders unable to respond to current voices and crises. What is necessary to interrupt the projection is to face ambivalence, to acknowledge one’s own destructiveness instead of locating all destruction in remote spaces and past times. This is prerequisite to encountering not solely a function of one’s own psychic organization but to facing an actual counterpart, or adversary, and responding to them.

Still, the traumatic projection in Fritsch’s text is no mere lapse, but mirrors a structural problem. A critical response to the ongoing transmission of discourses that justify, foster, excuse, or deny mass violence is always necessary but particularly challenging with regard to Eastern Europe, as Stepanova’s essayistic novel Памяти памяти (In Memory of Memory) points out. The text portrays the difficulty of articulating a commemorative familial narrative of the author’s Jewish Russian ancestors in the context of a largely repressed communal memory of Nazi and Soviet terror. *In Memory of Memory* reports the process of reconstructing a family history based on an archive full of photographs, letters, and diaries discovered after the death of an aunt of the narrator. The text portrays the fate of family members in critical moments of Russian and European history and points out the many unknowns which accompany the known episodes of the past. What sets Stepanova’s text apart from the large corpus of contemporary family history narratives is that it is conscious of how easily personal memory narratives tie in with legitimizing the neoliberal status quo by presenting a much more horrible past. Stepanova addresses this issue by reverting the common order of critical discourse as the literary text discusses theoretical concepts of memory studies which have been formed in reading literary texts, most notably the notion of “postmemory”. ^52^ “Postmemory” does not imply an end of memory but, rather, the “ *structure* of inter- and transgenerational transmission of traumatic knowledge and experience” ^53^ onto descendants who cannot have a personal recollection of the traumatizing events themselves. The “generational remove” ^54^ in traumatic memories can be regarded as one of the reasons for a renewed, and altered, interest in complicity with Nazi perpetration, Soviet terror, or other instances of twentieth century mass violence and totalitarianism in current literatures: generations who have not witnessed the past violence themselves approach it against the backdrop of their own involvements. However, in Central and Eastern European audiences, there is often a lack of a clear “generational remove”, as Stepanova suggests, so that complicities with political violence and involvement in neoliberal wrongdoing do, in fact, converge in individual experience. Stepanova’s In *Memory of Memory* challenges the Western bias of memory studies that the transgenerational transmission of trauma anachronistically imports psychosocial sequelae of past violence into a present that is profoundly different from that past. For political and socioeconomical realities often remain unchanged even after the demise of terror regimes. ^55^ The experience of totalitarianism and political (mass) violence that spread over several generations also means that, as Sindbæk Anderson and Törnquist-Plewa outline, “[c]ategories such as victims, perpetrators, collaborators and bystanders, often used in the Western discourse about World War II, are very difficult to apply” as individuals and groups have often “shifted their roles with the many, often violent, turns in the history” of the realm. ^56^ Consequently, Stepanova’s narrator traces her family’s involvements from a peculiar point of view:

In Russia, where violence circulated ceaselessly, society passing from one space of tragedy to the next *as if it were a suite of rooms, a suite of traumas*, from war to revolution, to famine and mass persecution, and on to new wars, new persecutions—the territory for this hybrid memory formed earlier than in other countries (…). ^57^

The Russian original speaks not of “a suite of traumas,” but of a traumatic enfilade, травматическая анфилада. ^58^ The French *enfilade* denotes a suite of rooms where all connecting doors are aligned in a single axis. Crucial for Stepanova’s text is that the suite can only be seen from a point of view aligned with that axis, not from outside the alignment. Enfilades are part of feudal architectural grandeur. The notion of a “traumatic enfilade” suggests that starting with czarist despotism, the sequence of historical events in Russia and the Soviet Union in the ninetieth, twentieth and twenty-first centuries have created the point of view from where an observer cannot claim to look *back* at a distanced past but speaks from within an ongoing transmission of terror and violence that requires critical reflection in order not to participate in the reproduction of sequelae of victimization, perpetration, and complicity.

Stepanova’s text folds analytical terminology back into the literary text because she doubts the two fundamental claims of conceptual language: distance and generalization. Insisting on analytical distance misses the seminal, albeit uncomfortable affectedness and involvement of the observer that is the very reason for analysis and criticism. Generalizing memorial structures found in Western Europe contributes to hiding this affectedness. From a non-Western point of view, understanding the transgenerational transmission of trauma and psychosocial sequelae of perpetration is necessary because the violence inducing them is not strictly past. This intervention does not run counter to the conceptual foundations of memory studies. Trauma, as well as discourses that justify terror and mass violence, undermine the commonsensical temporality of before, during, and after the fact—the latter in that they often set the linguistic and heuristic frame for the subsequent moral and juridical evaluation of violence to the effect that they are not strictly past but last. A striking case in point is, as discussed above, the concept of “totalitarianism”, which had been seminal to the rhetoric of Fascism and National Socialism ^59^ and is, still, a technical term of political science—a concept employed, not least, to differentiate an economically detrimental form of rule from economically productive authoritarianism. ^60^

Such unwitting transmission of justifications of mass violence is a result also of the second strategy of distancing I want to highlight.

## Moral distancing

There can be no doubt that mass violence deserves moral condemnation. What I call moral distancing (for the time being, for lack of a better term) is the strategy to secure the author’s and the reader’s non-involvement in the portrayed acts of mass violence and totalitarian terror by casting them as morally, and often also intellectually, superior to portrayed complicit characters. This moral high ground is problematic because is presumes that there is no ongoing transmission of discourses which justify, foster, excuse, or deny perpetration or complicity, which allows audiences to consume a supposedly disconnected, horrific past as exotic alterity. However, the very presumption of profound moral distance—that “it can’t happen here” or now—is a seminal precondition for transmitting such discourses, ^61^ notably as the consumption of the past as exciting entertainment is prone to aesthetically reproduce victimization by turning suffering into a spectacle.

Yet even the display of traumatic memories in shocking rather than consoling images still poses the same problem, as recent criticism holds:

trauma is placed in a marketplace […], being revalued in each transaction according to the logic of supply and demand. Victim and witness; witness and reporter; reporter and audience; producer and consumer: all these parties bargain to suit their different interests. ^62^

The role of the accomplice in memorialization and in the commodification of suffering that is missing from the above list is the focus of Jelinek’s 2008 play *Rechnitz (Der Würgeengel)* (trans. Rechnitz [The Extermination Angel], 2015). Jelinek’s text portrays the transmission of local knowledge about a 1945 massacre near the Austrian town of Rechnitz, close to the Hungarian border, where a few days before the arrival of the Red Army, at a dinner party hosted by Margit von Batthyány, daughter of Heinrich Thyssen at the local castle, 180 to 200 Hungarian Jews were shot by party guests. Most of the victims’ bodies have not been found to this day, and the known perpetrators have evaded prosecution. The 1994 documentary film *Totschweigen (Wall of Silence)* ^63^ portrays an unsuccessful campaign to recover the bodies as well as the locals’ reports on the event. A local hunter points out one of the many assumed sites of the massacre and the mass grave, and comments on it: *Also wir befinden uns, würde ich sagen, auf dem Platz, wo sich die Tragik in etwa zugetragen hat.* (“We are on the site, I would say, where the tragedy approximately happened.”) ^64^ Pointing upward, he later closes: *Wenn‘s jetzt da nicht ist, und nicht gefunden wird, dann sollte man die Sache mehr oder minder dem Höheren überlassen und endlich, möchte ist fast sagen, Ruhe geben.* (“If this turns out not to be the place, and it is not found, one should more or less leave the affair to the Almighty and finally, I would almost say, be quiet.”) ^65^ This is a striking illustration of what it means for perpetrators, accomplices, and their descendants to call mass violence a tragedy: it means *not* calling it murder, casting it as rule of a superior force (so that no one is a responsible actor with intent), and implying that the victims were guilty of some hubris punished by their ‘tragic fate’ (which is not only the logic of Attic tragedy, but also a key element of antisemitism).

Jelinek’s play references the documentary film as one of its sources but desists from giving a clear account of the massacre itself and thus responds drastically to the question of whether the representation of past violence should become a source of entertainment or consolation for the spectator. This issue is by no means detached from understanding historical massacring which is, as historian Sémelin puts it, “the most spectacular practice which those in power have at their disposal to assert their ascendancy”. ^66^ If this is true, then massacre wants to be watched. In order not to assume a position in this order of violence, not to confirm the logic of massacre by way of mimetic participation, Jelinek focuses on the local testimonies, on the cruelty and denial that is not past but still passed on by a multitude of voices that seem to testify to events while claiming their personal non-involvement:

*Ich als Bote hätte Ihnen selbstverständlich gern einen Beweis in Gestalt eines Zeugnisses gegeben, aber dadurch wäre ich ja Zeuge geworden, nicht Bote, und hätte mich womöglich selber strafbar gemacht, weil ich sowas auch nur mit angesehen hätte, wobei die Betonung auf mit liegt, denn es waren auch andre da, […] und zwar Leute, die angesehener waren als Sie!* ^67^

As a messenger I would have certainly liked to provide you with more reliable written records, but that would have made me a witness and possibly liable for prosecution, just because I might have seen what others saw and the emphasis is on others, there were others who saw what I saw, in plain sight even of the Holy See. So what do you want from me? ^68^

The official English translation of this passage differs slightly from the German original in that it harps on an English homonym to link the act of *see*ing to the inactivity of the Holy *See*, i.e., Pope Pius XII. (The actual etymology of “Holy See” is, of course, Latin *sedes*, “seat”.) The German text, rather, links the act of seeing ( *sehen*) to the respectable ( *angesehen*) status of the many inactive witnesses. Thus exploring what follows from seeing, i.e., testifying, *Rechnitz* can be said to establish a poetical distinction akin to the terminological distinction drawn by Sanders ^69^: between, on the one hand, “responsibility-in-complicity,” i.e., how her texts responds to violence, namely by not portraying it as source of amusement and, on the other hand, “acting-in-complicity” staged by actors. Their complicity lies in the way they fulfill their role as messenger. Their aim is not to reveal what happened out of plain sight as in tragic or Biblical messengers but, rather, to prevent clarity while also enjoying the importance of the role of the messenger. This requires not telling anything while also talking all the time, indicating just enough to make clear that there is a secret to uncover:

*[…] ich könnte Ihnen noch sagen, wo kein leichtes Finden dieser Leichen sein wird, kein leichtes Finden wird das sein, aber ich sage nichts, sonst wäre es ja ein leichtes Finden und ein viel zu leichtes Finale. ^70^*

“I could also tell you where it will not be easy to find those bodies, no, nothing will be easy to find, but I say nothing, otherwise it would be too easy a find and much too easy a finale.” ^71^

Focusing on messengers rather than historical events, Jelinek’s poetics of anonymous voices “performs”, as Grobbel writes with reference to earlier plays by Jelinek, “those complicit practices and ways of thinking that bring about cultural politics of exclusionary and annihilating violence.” ^72^ Jelinek’s focus on the transmission of justificatory discourses, however, also reflects on the distancing implied in the gesture of finding others complicit in mass violence and totalitarianism—a distancing the film allows, too. For it is easy to dismiss the pictured gentleman as old Nazi, to feel morally and intellectually superior to him. The distancing effect of this moral high ground is the basis of Eva Menasse’s 2021 novel *Dunkelblum*, which is based on the Rechnitz massacre without spelling out the name. ^73^ The narrative positions in Menasses’ texts are clear-cut and free from all ambivalence when a third-person omniscient narrator sorts out few good characters among many parodistic bad ones.

*Die Leute sprachen ihre Sätze oft nicht zu Ende, bei solchen Themen eine gute Strategie. Man deutete an, was man dachte, und hatte doch nichts gesagt. Danach gab es einiges Gelächter und ein paar schmutzige Witze […] In Wahrheit lauerten sie auf Neuigkeiten und waren ungewiss, ob hier mit etwas Ärgerlichem oder Sensationellem zu rechnen war*. ^74^
“People often didn’t finish their sentences, a good strategy with such topics. One hinted at what one thought and, still, had not said a thing. Later, there was some laughter and a few dirty jokes […] In truth, they were on the lookout for news, unsure whether to reckon with something annoying or sensational.”

The reader is invited to share the narrator’s moral superiority of knowing “in truth” that the novel’s characters are voyeuristic—and invited to deflect his or her own pleasure in reading about them, and the detective story of uncovering atrocities. Jelinek’s text impedes such complacent pleasure by questioning the role of the audience in the portrayal of historical acts of mass violence. Why watch, or read—in short: consume—the reproduction of this desolate speech of repression and denial? To create distance, one of the text’s voices suggest: *Wir lagern Gut und Böse aus, dorthin, wo wir nicht sind. So gehört es sich, das nennt man Outsourcing* ^75^ (“We transfer good and evil to where we are not. That’s how it’s done, it’s called outsourcing.”) ^76^ The identification of perpetrators and accomplices creates the good conscience of not being guilty oneself, the consolation of knowing who is, and it also allows the campaign to recover the massacre’s victims to turn into a narrative of suspense.

In order to highlight the role of the spectator and the involvement of the messenger in transmission, Jelinek intensively references a lesser-known Greek tragedy, Euripides’ *Bacchae*, which relies heavily on messengers to convey the gruesome plot on stage: people being torn apart and eaten alive by the hands and mouths of women frenzied by Dionysus, god of theatre. Dionysus elicits an interesting confession from his antagonist eager to catch sight of these women:

DIONYSUS: Ah! Do you wish to see them sitting together on the mountains?
PENTHEUS: Very much so, and would give an enormous weight of gold to do so.
D: Why have you fallen into a great passion [lust: ἔρως] for this?
P: It would give me pain to see them drunk.
D: And yet you would enjoy seeing things that are bitter to you?
P: To be sure, sitting in silence under the firs. ^77^

Pentheus wants to see, not in spite of, but because of being disgusted by the sight. Yet the intradiegetic exchange also pertains to the metadiegetic level, to the spectators of the play in the audience. They are far from uninvolved, distanced, “sitting in silence under the firs”. They are eminently implicated because their gaze, affectedness, and emotional response is the very purpose of the theatrical performance. Euripides highlights the voyeurism implicit in the stance of the spectator of a staged play, the lust of looking at violence from a supposed distance. Jelinek references Euripides’ tragedy among her sources and cites it in quotes and allusions, pointing out that literary genres comprise a reflection of the stance and participation of their audience. In referencing Euripides’ play, which emphasizes the complicity of the theater audience, Jelinek highlights that her documentary fiction on stage is not a genre of moral purification, but one of contamination. She desists from staging the massacre, and thus from turning the past violence into a source of present enjoyment, in order to draw attention to the audience’s participation in violence—a—participation that Sigmund Freud sees inscribed in the staging of victimization: tragedy seems to provide spectators with the position of an uninvolved, distanced beholder but their gaze and pleasure—the purpose of the spectacle—makes them complicit in the violence. ^78^ Jelinek’s play highlights the lust that drives participation in the audience, the messengers and the party guests who appear to have committed a massacre for entertainment. This lust is pointed out by one of the messengers, who adopts the charge of complicity for the discourse of repression:

*Ich weiß ja, daß Sie von der Geschichte dieser schrecklichen Zeit geradezu hypnotisiert sind, Sie interessieren sich für gar nichts anderes mehr, das sehe ich, und indem Sie auf da Entsetzliche starren, das dieses Land verbrochen hat, machen Sie Deutschland wieder zum Nabel der Welt! ^79^*

“I know you are mesmerized by stories of those horrible times, you are not interested in anything else, I can see that, and by staring at the horrible crimes this country committed, you once again make Germany the navel of the universe!” ^80^

This passage makes clear that the charge of complicity is no means for producing clarity. It’s unsettling volatility that lends itself to very different agendas, rather, marks involvements that remain concealed in complacent ascriptions of perpetrator roles. The point of Jelinek’s documentary play is thus not least the ambivalence of its genre: documentary fiction creates attention for the suffering of victims, for the issue of participatory violence, and of complicity in the transmission of justificatory discourses, by making the audience participate in this very complicity.

## Conclusion

Why the interest in past complicities now? Recent Central and Eastern European literary texts point out that declaring problematic involvement in mass violence and totalitarianism *past*, such as in memorializing them as ‘tragic past’, functions as an efficient way of concealing the historical transmission of their respective justificatory discourses as well as their adoption in very different contexts in the interest of repressing and denying current involvement in neoliberal wrongdoing.

## Data availability

No data are associated with this article.

## Ethics and consent

Ethical approval and consent were not required.
