# Supplementary material for: Complicity at a distance: commemorating problematic involvement in perpetration in contemporary Central and Eastern European literatures
Source: Open Res Eur. 2022 May 25;2:42. Originally published 2022 Mar 25. [Version 2] doi: 10.12688/openreseurope.14631.2 (PMC10446075; doi:10.12688/openreseurope.14631.2)
Supplement: Peer review history

# Reviewer report: Reviewer response for version 2

**DOI:** 10.21956/openreseurope.16040.r29363

**Published:** 2022-06-27

**Authors:** Tobias Heinrich

**Affiliations:** 1 School of Cultures and Languages, University of Kent, Canterbury, UK

All reviewers' comments have been carefully responded to and this version is ready to pass peer review.

If any, are all the source data and materials underlying the results available?

No source data required

Is the work original in terms of material and argument?

Yes

Is the work clearly and cogently presented?

Yes

Is the argument persuasive and supported by evidence?

Yes

Does the research article contribute to the cultural, historical, social understanding of the field?

Yes

Does it sufficiently engage with relevant methodologies and secondary literature on the topic?

Yes

Reviewer Expertise:

NA

I confirm that I have read this submission and believe that I have an appropriate level of expertise to confirm that it is of an acceptable scientific standard.

# Reviewer report: Reviewer response for version 2

**DOI:** 10.21956/openreseurope.16040.r29364

**Published:** 2022-06-08

**Authors:** Magdalena Zolkos

**Affiliations:** 1 Department of Social Sciences and Philosophy, University of Jyväskylä, Jyväskylä, Finland

I approve the revised version of this article as ready to pass peer review.

If any, are all the source data and materials underlying the results available?

No source data required

Is the work original in terms of material and argument?

Yes

Is the work clearly and cogently presented?

Yes

Is the argument persuasive and supported by evidence?

Partly

Does the research article contribute to the cultural, historical, social understanding of the field?

Yes

Does it sufficiently engage with relevant methodologies and secondary literature on the topic?

Yes

Reviewer Expertise:

NA

I confirm that I have read this submission and believe that I have an appropriate level of expertise to confirm that it is of an acceptable scientific standard.

# Reviewer report: Reviewer response for version 2

**DOI:** 10.21956/openreseurope.16040.r29365

**Published:** 2022-05-27

**Authors:** Johannes Kaminski

**Affiliations:** 1 Slovak Academy of Sciences, Bratislava, Slovakia

This is an excellent piece of research, I have no further comments.

If any, are all the source data and materials underlying the results available?

No source data required

Is the work original in terms of material and argument?

Yes

Is the work clearly and cogently presented?

Yes

Is the argument persuasive and supported by evidence?

Yes

Does the research article contribute to the cultural, historical, social understanding of the field?

Yes

Does it sufficiently engage with relevant methodologies and secondary literature on the topic?

Yes

Reviewer Expertise:

My areas of expertise are World Literature, Science Fiction, Goethezeit, Chinese literature

I confirm that I have read this submission and believe that I have an appropriate level of expertise to confirm that it is of an acceptable scientific standard.

# Reviewer report: Reviewer response for version 1

**DOI:** 10.21956/openreseurope.15800.r28999

**Published:** 2022-05-18

**Authors:** Magdalena Zolkos

**Affiliations:** 1 Department of Social Sciences and Philosophy, University of Jyväskylä, Jyväskylä, Finland

This article gives an overview of selected literary works that narrate instances of historical complicity in the Central East European contexts, and makes a series of original observations about the prevalence or a 'wave' of such literary representations in the 21st century European literature. The article anchors its detailed analyses of two literary texts in a theoretical and philosophic literature in memory and post-memory studies, trans-generational trauma theory, and testimonial theory (to mention just those). The concept of complicity is well outlined and the argument for the importance of literary accounts of complicity (by way of addressing structural and conceptual limitations of the juridical discourse on complicity) is persuasive. I have also enjoyed and learnt from the author's eloquent analyses of Jelinek's *Rechnitz *and Fritsch's *Hertzklappen*.

 Hence, the problem with the article is not the content or its conceptualisation, but, rather, its framing as presented in the opening sections. In my view, the article promises to do something else than it actually does - the opening questions about the present-day cultural traction of literary representations of complicity and its purport to the contemporary reader are not answered. Rather, the analytic focus throughout is on the aesthetic strategies of distanciation, and how these overlap and imbricate with the politics and ethics of (self) separation from difficult history. The opening point about what the author calls 'convergence'  (between historical complicity and the complexities of participation in exploitative economic, social and economic structures today) thus disappears in the main section of the article. My suggestion would be to re-frame the article so that it starts off with articulating the problem that it actually addresses and persuasively argues - how selected works of literature replicate and implement aesthetics of patio-temporal and moral distanciation, and how other literary works subvert and resist them.

 I also wonder (though could be convinced otherwise) whether this arrangement of two primary analyses (Fritsch and Jelinek) isn't somewhat too binary and too oppositional - one text is clearly presented as replicating problematic aesthetics of distanciation and the other as subverting them. In this binary, the references to Stepanova's *In Memory of Memory* are pushed to the periphery of the paper, but the author's insight is fascinating and left me wanting to read more. Perhaps it would be possible to give a more prominent place in the article to Stepanova so that there is a detailed analysis of three literary works, and not two, and the author explored how the subversion or 'undoing' of the strategies of distanciation happens in Stepanova? It might be an issue of a word count to extend the paper in this way, but might be worthwhile to consider. 

 Finally, I also want to point to a number of places in the paper where interesting formulations are somewhat underdeveloped and could benefit from more elaboration, or where turns of an argument are too abrupt:

- The reference to the point on 'totalitarianism' on pp. 7 & 8 comes and ends too abruptly; could it be folded more onto the analysis?
- The reference to Bacchae, and its relevance for the author's analysis of Jelinek needs more development; it intrigues, but its significance is somewhat elusive.

For the discussion of Arendt's point from *Responsibility and Judgment*, see also Arendt's rather little-discussed short text "Answers to Questions Submitted by Samuel Grafton" (included in *The Jewish Writings* collections ed. by Kohn and Feldman), where she develops the point on guilt and responsibility. ^1^

If any, are all the source data and materials underlying the results available?

No source data required

Is the work original in terms of material and argument?

Yes

Is the work clearly and cogently presented?

Yes

Is the argument persuasive and supported by evidence?

Partly

Does the research article contribute to the cultural, historical, social understanding of the field?

Yes

Does it sufficiently engage with relevant methodologies and secondary literature on the topic?

Yes

Reviewer Expertise:

literary trauma criticism, theories of witnessing

I confirm that I have read this submission and believe that I have an appropriate level of expertise to confirm that it is of an acceptable scientific standard, however I have significant reservations, as outlined above.

## References

[1] : Answers to Questions Submitted by Samuel Grafton in The Jewish Writings ed. J. Kohn, R. H. Feldman. *Schoken* .2007;

# Response

**Published:** 2022-05-20

**Authors:** Juliane Prade-Weiss

**Affiliations:** Ludwig Maximilian University of Munich, Germany

I thank all reviewers for their detailed reading od my paper, their encouraging words, and their very useful criticism that was critical to improving the argument!

 I have addressed all concerns, which means, in detail: The most important point of criticism shared by all reviewers was a certain gap between the lead hypothesis on convergences between complicities in totalitarianism and involvement in neoliberalism on the one hand, and the focus on distancing on the other hand. I have addressed this absolutely right observation by:

1. Improving the framing so as to differentiate the comprehensive background of my reading from the element of that background that can actually be addressed in a paper (see Introduction).
2. Added a substantial segment to the part “Complicity in and beyond the Law” explaining how the broader frame and the question of distance correlate, and I come back to it in paragraphs added to the readings of Fritsch and Jelinek.

Further, smaller points have been addressed as follows:

- I have expanded the signposting explaining why I juxtapose Fritsch’s and Jelinek’s text, and I have added a note to my detailed discussion of Stepanova elsewhere (which is the reason why I did not expand it is this paper).
- I have, as noted in the general point, substantially expanded the discussion of totalitarianism. I have expanded the explanation of the reference to Bacchae to explain how it relates to the issue of complicity and distance.
- I am also thankful for making me aware of Arendt’s text of her Eichmann book. However, I have not included it because a) the discussion of guilt and responsibility of Eichmann refers to an individual rather than the relational aspect I want to focus on, and b) the tremendously difficult question of the Judenräte discussed in Arendt would require a detailed discussion that would go beyond the scope of my article. But I will certainly come back to the text in my further work.

# Reviewer report: Reviewer response for version 1

**DOI:** 10.21956/openreseurope.15800.r28998

**Published:** 2022-04-27

**Authors:** Tobias Heinrich

**Affiliations:** 1 School of Cultures and Languages, University of Kent, Canterbury, UK

This article explores the reflection on complicity in terror and perpetration across Eastern and Central European literatures. It is highly original in its operationalisation of complicity, which is first and foremost presented as a legal concept, in a narratological context. The analysis is particularly convincing where it demonstrates how literary narratives can involve readers, but also offer ways of distancing them from the perpetration that the texts refer to. Examples like Valerie Fritsch' *Herzklappen von Johnson & Johnson *or Eva Menasse's *Dunkelblum* demonstrate how narrative strategies of spatial, temporal and moral distancing create an effect of reassuring consolation for the reader, where a text addresses collective complicity in atrocities committed within totalitarian rule. Finally, the author shows how Elfriede Jelinek's *Rechnitz (Der Würgeengel) *consciously avoids such consolation as it exposes how readers / the audience themselves become complicit when they voyeuristically enjoy the narrative engagement with atrocities of the past.

 Similar to the first peer reviewer, I believe that the article is convincing in its main points, but the argument about the reasons for the current rise in interest about atrocities during Nazi and Soviet rule needs to be rethought. It features prominently in the abstract, the introduction and the conclusion, but is not sufficiently addressed in the main part of the article. Generally, the text is quite ambitious despite it being a relatively short essay of roughly 5000 words. While the general concept is strong, some of the examples for distancing effects are only presented very briefly and a more extensive discussion of the literary texts the author refers to would help to carve out these ideas more clearly.

If any, are all the source data and materials underlying the results available?

No source data required

Is the work original in terms of material and argument?

Yes

Is the work clearly and cogently presented?

Yes

Is the argument persuasive and supported by evidence?

Yes

Does the research article contribute to the cultural, historical, social understanding of the field?

Yes

Does it sufficiently engage with relevant methodologies and secondary literature on the topic?

Yes

Reviewer Expertise:

literary criticism, narratology, life writing, German literature from 1700-present

I confirm that I have read this submission and believe that I have an appropriate level of expertise to confirm that it is of an acceptable scientific standard, however I have significant reservations, as outlined above.

# Response

**Published:** 2022-05-20

**Authors:** Juliane Prade-Weiss

**Affiliations:** Ludwig Maximilian University of Munich, Germany

I thank all reviewers for their detailed reading of my paper, their encouraging words, and their very useful criticism that was critical to improving the argument!

 I have addressed all concerns, which means, in detail: The most important point of criticism shared by all reviewers was a certain gap between the lead hypothesis on convergences between complicities in totalitarianism and involvement in neoliberalism on the one hand, and the focus on distancing on the other hand. I have addressed this absolutely right observation by:

1. Improving the framing so as to differentiate the comprehensive background of my reading from the element of that background that can actually be addressed in a paper (see Introduction).
2. Added a substantial segment to the part “Complicity in and beyond the Law” explaining how the broader frame and the question of distance correlate, and I come back to it in paragraphs added to the readings of Fritsch and Jelinek.
3. I thank Johannes Kaminski for making me aware of Biebricher’s study on the history of neoliberalism and his argument, which I also included.  

Further, smaller points have been addressed as follows: I have added a section of further possible reasons for the current rise in interest in Nazi and Soviet atrocities to the discussion of Stepanova’s text. I have thus expanded the discussion of her text, and also of Fritsch’s and Menasse’s text, notably by adding my translation (as their books have not been translated yet).

# Reviewer report: Reviewer response for version 1

**DOI:** 10.21956/openreseurope.15800.r28926

**Published:** 2022-04-06

**Authors:** Johannes Kaminski

**Affiliations:** 1 Slovak Academy of Sciences, Bratislava, Slovakia

The article inquires into the functions of contemporary documentary fictions that engage with the horrors of the Third Reich and Soviet Union. By drawing on a legal category, complicity, the author offers a highly original approach to evaluating ethical attitudes implicit in documentary fiction. While the concept occupies a marginal position in legal discourse, complicity becomes analytically fertile when applied to the realm of fictional settings that shape the collective memory.

 The author’s case examples include Jachym Topol’s *The Devil’s Workshop*, Valerie Fritsch’s *Herzklappen von Johnson & Johnson*, Maria Stepanova’s *In Memory of Memory* and Elfriede Jelinek’s *Rechnitz*, the main focus being placed on Fritsch and Jelinek. Accordingly, Fritsch’s text, irrespective of its poetic qualities, exemplifies an ethical stance that problematically continues the aggressor’s perspective. During the narrator’s journeys through Eastern Europe, its territories remain faceless places deprived of socio-political agency. The author’s strong criticism of Fritsch is supported by enlightening references to Stepanova. One can only agree with the author’s argument. The text’s narrator keeps a safe distance from the events of the past, a stance that is afforded by the elision of the East’s agency and present-day reality.

 By way of contrast, the author singles out Jelinek’s play as a glowing example of a more truthful engagement with the past. In her account of the Rechnitz massacre, justificatory discourses take centre stage. Rhetoric cliches are not only instrumental in deflecting guilt, but are intrinsically linked to a questionable aesthetics of dramatic jouissance, originating in Greek antiquity. While the reviewer does not agree with the author’s idea that Jelinek’s drama lacks a self-righteous “moral high ground”, this benevolent assessment is well-accepted in academe and does not need any further explanation.

 The only reservation concerns an idea that is, although prominent in the abstract, only faintly sketched in the main argument: the hypothesis “that the texts address convergences between involvements in past acts of mass violence and current forms of participation in wrongdoings in neoliberalism” (p. 1). In this argument, the distinctions between totalitarianism and neoliberalism are flattened through a sweeping generalisation: Since both forms of government require the individual's complicity in oppression, argues the author, totalitarian and neoliberal modernity must be considered continuities. (The critical reference to this notion is Dupuy 2016.) Even in Neomarxist thought, this makes for an unusual argument, as it goes against basic assumptions in political science. In fact, classic liberalists like Hayek regard the welfare state as more closely linked to totalitarianism than neoliberalism. (See Thomas Biebricher, *The Political Theory of Neoliberalism*, Stanford: Stanford UP, 2018, pp. 18–21.) While nothing speaks against expanding on this provocative statement, it would require substantial discussion. This argument is not central to the paper. For the sake of clarity, the author should either elaborate on this argument or remove it.

 Marginalia:

 P. 7: “Marcon” > “Macron” (7).

 P. 9: Although the author is undoubtedly aware of the etymology of “Holy See”, the text suggests that the term is related to “seeing” (Engl.) rather than “sedes” (Latin).

 Footnotes 1 & 15: The authors is advised to double-check quote marks in the footnotes, as German and English usage is mixed.

If any, are all the source data and materials underlying the results available?

No source data required

Is the work original in terms of material and argument?

Yes

Is the work clearly and cogently presented?

Yes

Is the argument persuasive and supported by evidence?

Yes

Does the research article contribute to the cultural, historical, social understanding of the field?

Yes

Does it sufficiently engage with relevant methodologies and secondary literature on the topic?

Yes

Reviewer Expertise:

My areas of expertise are World Literature, Science Fiction, Goethezeit, Chinese literature

I confirm that I have read this submission and believe that I have an appropriate level of expertise to confirm that it is of an acceptable scientific standard, however I have significant reservations, as outlined above.

## References

[1] : The Political Theory of Neoliberalism. *Stanford University Press* .2019;

# Response

**Published:** 2022-05-20

**Authors:** Juliane Prade-Weiss

**Affiliations:** Ludwig Maximilian University of Munich, Germany

I thank all reviewers for their detailed reading of my paper, their encouraging words, and their very useful criticism that was critical to improving the argument!

 I have addressed all concerns, which means, in detail: The most important point of criticism shared by all reviewers was a certain gap between the lead hypothesis on convergences between complicities in totalitarianism and involvement in neoliberalism on the one hand, and the focus on distancing on the other hand. I have addressed this absolutely right observation by:

1. Improving the framing so as to differentiate the comprehensive background of my reading from the element of that background that can actually be addressed in a paper (see Introduction).
2. Added a substantial segment to the part “Complicity in and beyond the Law” explaining how the broader frame and the question of distance correlate, and I come back to it in paragraphs added to the readings of Fritsch and Jelinek.
3. I thank Johannes Kaminski for making me aware of Biebricher’s study on the history of neoliberalism and his argument, which I also included.

I have also corrected the typos; I am very thankful they have been brought to my attention!
